# Peanut Allergenicity: An Insight into Its Mitigation Using Thermomechanical Processing

**DOI:** 10.3390/foods12061253

**Published:** 2023-03-15

**Authors:** Elissa Haidar, Jack Lakkis, Marc Karam, Mohamed Koubaa, Nicolas Louka, Espérance Debs

**Affiliations:** 1 Faculty of Medicine, University of Balamand, P.O. Box 100, Tripoli 1300, Lebanon; 2 Department of Biology, Faculty of Arts and Sciences, University of Balamand, P.O. Box 100, Tripoli 1300, Lebanon; 3 Université de Technologie de Compiègne, ESCOM, TIMR (Integrated Transformations of Renewable Matter), Centre de Recherche Royallieu - CS 60319, 60203 Compiègne CEDEX, France; 4 Centre d’Analyses et de Recherche, Unité de Recherche Technologies et Valorisation Agro-Alimentaire, Faculté des Sciences, Université Saint-Joseph de Beyrouth, Riad El Solh, P.O. Box 17-5208, Beirut 1104 2020, Lebanon

**Keywords:** peanut allergy, immunoreactivity, allergenicity mitigation, thermomechanical processing

## Abstract

Peanuts are the seeds of a legume crop grown for nuts and oil production. Peanut allergy has gained significant attention as a public health issue due to its increasing prevalence, high rate of sensitization, severity of the corresponding allergic symptoms, cross-reactivity with other food allergens, and lifelong persistence. Given the importance of peanuts in several sectors, and taking into consideration the criticality of their high allergic potential, strategies aiming at mitigating their allergenicity are urgently needed. In this regard, most of the processing methods used to treat peanuts are categorized as either thermal or thermomechanical techniques. The purpose of this review is to provide the reader with an updated outlook of the peanut’s allergens, their mechanisms of action, the processing methods as applied to whole peanuts, as well as a critical insight on their impact on the allergenicity. The methods discussed include boiling, roasting/baking, microwaving, ultrasonication, frying, and high-pressure steaming/autoclaving. Their effectiveness in alleviating the allergenicity, and their capacity in preserving the structural integrity of the treated peanuts, were thoroughly explored. Research data on this matter may open further perspectives for future relevant investigation ultimately aiming at producing hypoallergenic peanuts.

## 1. Introduction

Allergy is an adverse or augmented immunological reaction against certain substances that are basically supposed to be harmless. It is a sort of hypersensitivity launched when the body comes into contact with either natural or synthetic molecules. Four different types of hypersensitivity reactions exist. Types I, II, and III are antibody-mediated reactions, while type IV is a cell-mediated reaction. Symptoms of the first three types appear within minutes or hours after exposure to the allergen. Those of the fourth type, referred to as delayed-type hypersensitivity (DTH) reactions, require days or weeks. Most allergies are classified as type I, IgE-mediated and immediate allergic reactions; types II and III involve IgG or IgM and immune complexes, respectively. Estimated in about 10% of the population, food allergies must be tackled seriously and effectively due to, not only their detrimental physiological impact, but also their economic burden [1,2,3].

Peanuts, or *Arachis hypogaea* L., originated in South America. Labeled as “ground nuts” in the 1700s, they gained vast popularity as they were considered food for underprivileged families [4]. Peanuts are characterized by a rich nutritional content [5,6], represented by their macronutrients [5,6,7,8,9,10,11], micronutrients [5,8,10,11,12,13], and phytonutrients. Lipids, proteins, and carbohydrates constitute on average 50%, 26%, and 22% of the total mass of a peanut, respectively. Regarding the health aspect, various studies have demonstrated the beneficial implications of peanut consumption. They may play a key role in blood pressure regulation [10], in lessening cardiovascular diseases, heart failure, and myocardial infarction incidence [14], and in reducing gallstones [13], obesity and type 2 diabetes [15], cancer [16], and liver disease [17].

## 2. Peanut Allergy: Prevalence, Persistence, and Severity

**Figure 1:**
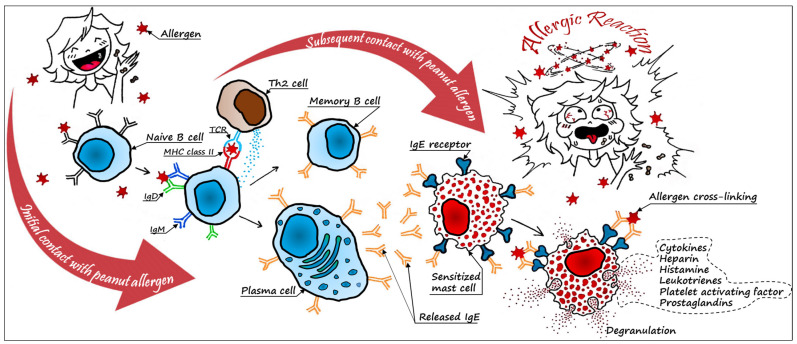
The mechanism of allergic reaction to peanuts.

Peanuts are notoriously known as allergenic legumes. Peanut allergy (PA) can be topical (cutaneous exposure or inhalation) or systemic. It garners massive attention since it is a nut that presents a prevalent, lifelong-persistent, and severe allergic reaction. Three distinct aspects characterize peanut allergy: the allergens expressed in the mature seeds, the sensitization phase, during which the susceptible individual will develop IgE antibodies against the proteins–allergens after the first exposure, and the elicitation phase occurring upon ingesting a sufficient eliciting dose of peanut allergens. Figure 1 illustrates the mechanism of allergic reaction. In atopic individuals, allergens pass to the bloodstream and tissues, inducing the production of IgE antibodies, which then bind to their receptors on basophils and mast cells. Consequently, the latter cells become sensitized (or primed) so that upon secondary exposure the allergen cross-links the bound IgE on the surface of the cells, leading to their degranulation. The released histamine and other allergy-causing factors provoke symptoms ranging from mild (skin rash, urticaria, pruritus, runny nose, edema, labored breathing, diarrhea, etc.) to life-threatening [3,18,19].

Elaborating on peanut allergy prevalence, more than 2% of people in Western countries are allergic to peanuts [20], including 1.8% of adults in the US [21]. An American longitudinal study revealed that PA incidence in one-year-old children increased from 1.7% to 5.2% between the years 2001 and 2017 [3,20], persisting in adulthood in most cases. A broad examination of all anaphylaxis admissions in the pediatric intensive care unit between years 2010 and 2015 (*n* = 1989) in the US, Mexico, and Canada, and another nationwide US examination of children (*n* = 38,308) confirm that PA was the most common trigger of severe reaction, where cases of PA anaphylaxis outweighed all other food allergies [20,22]. An analysis of 84 allergic children reported that 27% of them suffered from life-threatening symptoms following the first allergic reaction [20], and 71% of them had serious fatal complications at their second exposure. Out of the 73% who expressed non-life-threatening symptoms, 44% of patients’ lives were in danger after the second allergic reaction [19,20,22].

Allergenicity and IgE reactivity are determined by the interplay of a protein’s digestibility, solubility, stability, structural features, heat resistance, and enzyme activity, in addition to post-translational and chemical modifications [23,24]. On the other hand, several risk factors contribute to the increased prevalence of peanut allergenicity by amplifying its intensity and its spread. These factors may include genetics [25,26], epigenetics [27], and some processing methods such as roasting or dry heating, but also unintended exposure. This fact represents one of the main motivations for research on how to mitigate peanut allergenicity, since measures taken are not always fully adequate. Inconsistent food labeling standards (precautionary allergen labels) or inadequate safety measures in public settings (e.g., restaurants, airlines, and schools) create an environment conducive to accidental ingestion of peanuts. In terms of numbers, 7% to 14% of peanut-allergy patients undergo accidental exposure to peanuts on a yearly basis, increasing the risk of allergic reactions and potential anaphylaxis. Recent recommendations issued by the National Academies of Sciences, Engineering, and Medicine (NASEM) elaborate on the importance of allergen information on food labels.

## 3. Peanut Allergens and Their Mechanisms of Action

The Allergen Nomenclature Subcommittee of the World Health Organization/International Union of Immunological Societies (WHO/IUIS) reported 17 protein allergens contributing to PA and named after the species [19,28]. Upon revealing the peanut genome sequence, various proteomic studies were carried out to determine the allergens’ profile, their levels and modifications, such as reverse-phase high-pressure liquid chromatography [29], and more recently liquid chromatography–mass spectrometry [30]. Peanut allergens are classified as major and minor allergens, depending on their contribution to the initiation and propagation of an allergic response in sensitive hosts. A major peanut allergen binds specific IgE in more than 50% of allergic patients; otherwise, it would be classified as a minor peanut allergen. Among these proteins, scientists define Ara h 1, Ara h 2, and Ara h 3 as being the major protein allergens. In addition to their strong IgE-binding capacity, they represent more than 80% of the total protein pool contained in peanuts [6,31]. Recent studies stated that Ara h 6 displays a great recognition by immunoglobulins in the serum of allergenic patients, and that its biochemical structure and properties show a great similarity to those of Ara h 2 [19]. Peanut allergens, as reported in Table 1, are categorized into different superfamilies and families based on their biochemical, structural and functional characteristics [19,23,32,33,34,35,36,37,38].

### 3.1. Major Peanut Allergen Characteristics

Ara h 1, only in its glycosylated structure, triggered the maturation of Th2 (T helper 2) cells [39]. It is composed of three identical monomers linked all together to form a homotrimer. It contains 125 T cell epitopes and 23 to 25 allergic epitopes, four of which are immunodominant since they are recognized in more than 80% of patients [19,40,41]. These epitopes are not exposed on the outer surface of the native Ara h 1 trimer, a fact that renders this protein less allergenic in its natural form, and more allergenic once denatured through specific processing techniques [42]. Ara h 2 is associated with the highest IgE-binding capacity among all peanut allergens, with more than 95% reactivity in the sera of allergic patients. It is a glycoprotein with eight residues of cysteine that contains 65 epitopes, three of which (epitopes 3, 6, and 7) are immunodominant. Its structure presents five alpha-helices joined together by four disulfide bridges, providing high heat and digestion resistance associated with severe allergic reactions [40,43]. This allergen possesses two isoforms: Ara h 2.01 and Ara h 2.02 [44]. The latter includes one IgE-binding site more than the former, rendering it more allergenic. Ara h 3 (Ara h 3.01) takes the form of a hexamer crystal with two trimer rings interacting in a face-to-face orientation. This globulin, made of five isomers, was identified in approximately 40% of patients [3,19,43]. Ara h 3 displays four allergic epitopes: two that are exposed on the molecule in its native structure, and two buried yet capable of being uncovered following processing or enzymatic digestion, thus intensifying the allergenicity.

### 3.2. Minor Peanut Allergen Characteristics

Even though minor allergens might be masked by complex matrices such as cookies, cakes, sauces, chocolate, or others, they still have the possibility to elicit severe allergic reactions. Ara h 4, also named Ara h 3.02, is an isoform of Ara h 3, sharing 95% to 98% of its sequence identity. Four T cell epitopes have been identified in each one of Ara h 3 and 4. Ara h 5 is formed by three weakly bonded alpha-helices and a seven-stranded anti-parallel sheet. Feeble bonds are the reason behind its weak resistance to heat and digestion [45]. Moreover, it is found in minute amounts in peanut protein, resulting in low-frequency sensitization in peanuts. Detected in around 13% of allergic patients, Ara h 5 exhibits similarity with the pollen allergen Bet v 2 protein. Ara h 6, a trypsin inhibitor, is similar to the secondary and tertiary structure of Ara h 2. They both contain four firmly coiled helical structures in their core, thus providing high resistance to heat treatment and digestion. Five of seven Ara h 2 IgE-binding linear epitopes are homologous to Ara h 6 by 70 to 93% [46,47]. Ara h 7 shares 53% similarity in its structure with Ara h 6. It has two conserved disulfide bonds that render it weaker and less stable after exposure to heat or digestion compared to Ara h 2 and Ara h 6. In a study of allergic patients (*n* = 40), Ara h 7 was recognized in 43% of them [19,40]. Ara h 8 has weak bonds with low allergenicity in most cases. It is a pathogenesis-related (PR) protein that causes allergic symptoms in the oral cavity: oral allergy syndrome (OAS). Ara h 9, Ara h 16, and Ara h 17 all have eight cysteine residues in common. They transfer lipids, phospholipids, fatty acids, and their derivatives across two media (e.g., membranes). They also play a defense role against pathogens (bacteria, viruses, and fungi). These lipid-transfer proteins (LTP) are of two types, mostly LTP1 long-chain molecule and LTP2 short-chain molecule, relatively holding allergenic properties. A serious allergic reaction is triggered due to their resistance to digestion, which can preserve the LTP structure up to the intestinal tract. Ara h 9 is mainly found in the sera of patients living in the Mediterranean region. Ara h 10, Ara h 11, Ara h 14, and Ara h 15, belonging to the oleosin group, have highly conserved seventy residues forming a central hydrophobic domain and hydrophilic amino and carboxylic termini with slight differences in the primary sequence among them. Oleosins are remarkable oil bodies that contribute to oleosome stabilization and play an enzymatic role in aiding in germination [19,28]. Ara h 12 and Ara h 13 are small cysteine-rich molecules with stable domains that act as defensins, as their superfamily name reflects, and have immune reactive properties. As defensins, they are associated with an anti-fungal activity [48] Lastly, recent studies declare Ara h 18-cyclopilin as a peanut allergen protein. It is a peptidyl–prolyl cis–trans isomerase protein, requiring further analyses in order to elucidate the biochemical characteristics and action.

**Figure 2:**
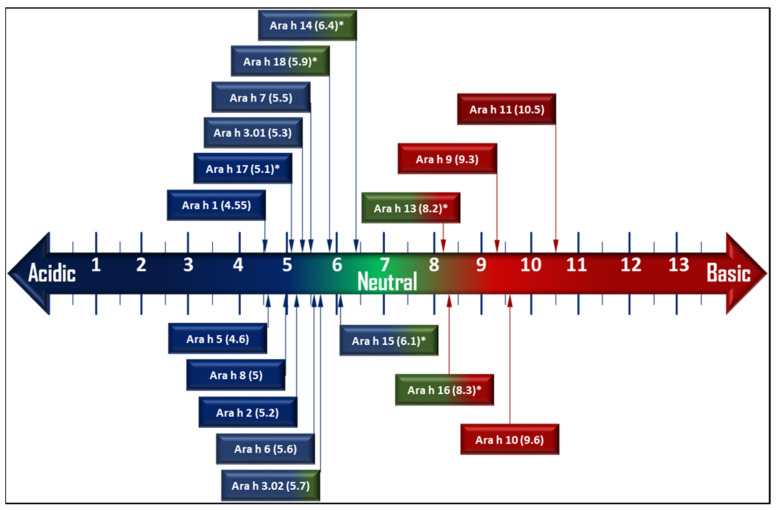
Distribution of pI of all peanut allergens on the pH scale. (pI values marked with * are estimations deduced from the literature studying SDS–PAGE of peanut allergens).

Finally, Figure 2 provides information about the pI (isoelectric point) of all peanut minor and major allergens. Some pI values were available in previous studies, and some others (marked with an asterisk) were calculated after SDS–PAGE (sodium dodecyl–sulfate polyacrylamide gel electrophoresis) used in the corresponding studies. Such charts may aid in a better understanding of the mode of action and residence of each allergen, paving the way for probable efficient methodologies for alteration.

### 3.3. Cross-Reactivity

Cross-reactivity is a distinctive feature of peanut allergenicity that increases its prevalence, thus impacting its clinical correlation. Patients sensitized to peanuts are at a higher risk of being sensitized to other nuts or foods, since all these allergens share many similar biomolecular patterns. More specifically, major allergens in legumes and tree nuts belong to the same limited number of protein families [24,43]. For instance, peanut cross-reactivity with soybeans is due to the similarity of peanut allergens Ara h 2 and Ara h 6 with Gly m 8, in addition to the homology of Ara h 1, Ara h 3, Ara h 5, and Ara h 8 with Gly m 5, Gly m 6, Gly m 3, and Gly m 4, respectively. The same applies to lupin seeds for which the allergens Lup an 1, Lup a vicilin, and Lup an 11S belong to the same protein family as Ara h 1 and Ara h 3. In addition, Ara h 5 and Lup a 5 are homologous. Pea allergens Pis s 1, Pis s 2, and Pis s 3 are homologous to Ara h 1, Ara h 3, and Ara h 9, respectively. Other examples concerning chickpea allergens cross-react with those of peanuts due to the homology of Ara h 2 and Ara h 6 with Cis a 2S, as well as the similarity between Ara h 1 and Ara h 3 and Cis a 11S. As for the allergens contained in lentils, especially Len c 1, Len c 2, and Len c 3, they are homologous to Ara h 1/Ara h 3, Ara h 5, and Ara h 9, respectively [28,43]. Furthermore, minor peanut allergens Ara h 16, 17, and 18 are similar to pollen allergens, particularly olive pollen in the case of Ara h 18 [35,40]. Functions of peanut allergens, their cross-reactivity and clinical relevance [19,28,32,33,35,45,46,49,50,51] are summarized in Table 2.

## 4. Thermomechanical Processing of Peanuts

The allergenicity of peanuts is closely related to the linear and conformational allergic epitopes of the indigenous proteins. Altering the structure of peanut allergens will result in downstream modifications in their physicochemical properties, thus modifying their immunoreactivity. The degree of alteration of the allergic potential of immunoreactive proteins in peanuts is strictly dependent on the processing conditions. Historically, peanut processing started by converting the whole peanuts into peanut butter or flour in an attempt to reach hypoallergenicity. Later studies demonstrated that these treatments, which violate the structural integrity of the nuts, could not produce hypoallergenic peanut derivatives. The current processing methods include physical, chemical, and biological treatments. They can be applied directly on whole nuts, on their derivatives such as cracked peanuts, peanut flour, peanut powder, or even on their earlier developmental stage such as in the case of genetic engineering and gene modification.

Physical methods encompass not only traditional cooking processes but also novel techniques that are heat-based, wave-based, high-pressure-based, or any possible combination. Such treatments include boiling, frying (shallow and deep), roasting/baking, microwaving, ultrasonication, and many more that are applied to whole peanuts. Thermo-pressure-based treatments can range from simple cooking under mild pressure to autoclaving and high-pressure processing. These methods have been shown to induce a certain level of alteration in the allergenic properties of whole peanuts.

From another perspective, a wide variety of chemical treatments are applied over peanut protein extracts to reduce their allergenicity. Chemical modifications that proved to be effective include glycosylation (covalent association of peanut proteins with saccharides), magnetic bead adsorption, and treatment with various acids such as tannic, caffeic, oleic, citric and acetic acids [52].

Biological methods are majorly based on fermentation, genetic engineering, and enzyme catalysis. The latter alters the allergens by hydrolytic proteases e.g., alcalase, pepsin, chymotrypsin and trypsin, or even using polyphenol oxidase [52]. Peanut protein allergenicity was entirely alleviated after microbial fermentation using Bacillus genus [53]. Acid-induced denaturation and proteolysis, Maillard reactions, and glycosylation accompanying this process may have contributed to this reduction. Genetic engineering constitutes a novel and advanced biological hypoallergenic treatment for peanuts. It includes various techniques that modify the entire legume crop in order to replace, mutate, or eliminate the expression of allergic proteins in the nuts.

The processing techniques that this review is focused on are based on post-harvest thermal or thermomechanical treatments of whole peanuts under conditions that preserve their structural integrity, summarizing all the studies published between 2017 and 2022. Every technique will be chiefly discussed in terms of its mode of action and its effectiveness in the production of hypoallergenic peanuts.

### 4.1. Boiling

Boiling is the immersion of food in water at a temperature approaching 100 °C under atmospheric pressure. While it is conducted for cooking purposes, boiling can be considered an efficient way to partially decrease sensitivity towards peanuts. Ingestion of boiled peanuts by sensitized mice led to a subtle etiology (e.g., weight loss, itching, and diarrhea) compared to the severe allergic reaction caused by raw peanuts [54]. Although the pathology examination revealed jejunum breakage and splenomegaly in sensitized mice fed boiled peanuts, it occurred in a milder manner than what was observed in the case of raw peanut feeding [54,55]. Thymic Stromal Lymphopoietin (TSLP) gene expression significantly contributes to the ignition of inflammatory immune responses. Its analysis in mice revealed that upregulation of the gene’s activity was steeper in response to raw peanut introduction compared to that of boiled peanuts.

A possible reason behind this discrimination in etiology and pathology could be the chemical structures modified after boiling. UV spectra analysis proved the alteration of all three major peanut allergens’ structures (Ara h 1, Ara h 2, and Ara h 3) [55]. At 280 nm, the absorbance values of these allergens were remarkably decreased in boiled peanuts compared to raw ones. Other studies emphasized the change in the surface hydrophobicity of Ara h 1 through the measured slight elevation in the hydrophobic index from 52 in raw peanuts to 89 in boiled ones [56], which characterizes the degeneration of the protein allergens in treated peanuts. The circular dichroism spectra (CD) analysis of the three major allergens depicts the alteration of their secondary structure after heat treatment [54,55]. The α-helices of Ara h 1 decreased by 11.04%, whereas β-sheets increased by 7.42%. The microenvironment of tryptophan decreased in the tertiary structure, causing it to congregate in the inner region [56]. This change in the functional structure may be caused by de novo aggregations in the protein due to boiling.

Changes in solubility were assessed by measuring the changes in protein content after boiling treatment under mimicked physiological conditions by regulating addition of gastrointestinal juice. Bicinchoninic acid (BCA) assay and SDS–PAGE detected a change in protein content ranging from 30% to 55% after treatment [57]. The total extractable allergen content out of the total peanut protein decreased from 71% in the untreated peanuts to 29% in the boiled peanuts [58]. Total Ara h 2 concentrations gradually decreased as the duration of boiling increased due to leaching into cooking water [59]. Protein digestion is a commonly used factor to evaluate allergenicity. In a simulated gastric fluid (SGF) experiment, boiled peanut allergens manifested low stability upon digestion [55]. For instance, Ara h 3 pepsin resistance was dramatically reduced in its acidic subunits [57,60].

Several experiments in mice and humans confirmed the efficiency of boiling in decreasing elicitation through IgE and IgG titers [55,61]. The IgE-binding capacity of Ara h 1 in boiled peanuts was significantly lower than in crude peanuts [56]. It was also noted that the degranulation i.e., β-hexosaminidase activity based on the RBL-2H3 cells model, was decreased. This might be due to uncomplimentary fitting of allergens after structure modification to IgE, or decreased IgE activation by the treated allergens [60].

### 4.2. Roasting/Baking

These two cooking methods are used interchangeably in the literature. Roasting and baking are carried out through the exposure of peanuts to dry heat: convection, conduction, radiation, or a combination. Roasting has gained a lot of attention over the last few years since it was discovered to be having hyper-allergic rather than hypo-allergic effects. Etiologically, scratching behavior and diarrhea were prominent in mice fed roasted or raw peanuts [54]. The weight of mice fed with roasted peanuts was the lowest among treatment groups. Pathologically, TSLP gene expression was upregulated in the roasted peanut group. In contrast to the boiled peanut group, jejunal breakage and splenomegaly were more prominent using roasted peanuts than raw [54,55].

Severe modifications in the structure of peanut allergens were observed after roasting. Circular dichroism spectra implied the modification of the original secondary structure of the allergens. It shows new, previously hidden amino acids in crude peanuts, on the surface: neoepitope development [55,62]. UV spectra analysis comparison revealed that the absorbance at 280 nm was higher after roasting. This explains the massive denaturation and alteration of secondary and tertiary structures of proteins by roasting. Dark and clear bands at the top of the separating gel were observed in SDS sample-buffer soluble fractions after heat treatment [58]. As a result of the hydrophobic interaction and covalent cross-linking, high-molecular-mass formation and increased aggregation were noted after passing through the dominant endothermic transition at temperatures above 80 °C [57]. This, in turn, was illustrated by the decreased Ara h 1 soluble fraction after roasting [58]. In another experiment, Western blots of roasted and raw peanut extracts showed high intensities of Ara h 2 and Ara h 8 bands; an ELISA test validated the high levels of those bands [63]. The total extractable allergen content decreased from 71% in raw peanuts to 21% in roasted peanuts [58]. Recently, Ðukić and collaborators investigated the post-translational modifications (PTMs) of peanut proteins as affected by roasting [64]. The most common PTMs observed were oxidation (met), formylation (Arg/Lys), hydroxylation (Trp), and oxidation or hydroxylation (Asn). Importantly, this study shed light on the structural alteration, and hence the digestibility, of peanut proteins occurring following roasting, as revealed by the proteomic profiling.

Not only was the structure of the protein modified, but also the concentration of allergens was multiplied after roasting. It was recorded that Ara h 1 concentration increased after roasting, ultimately in the presence of reducing sugars, resulting in a phenomenon called Advanced Glycosylation End Products (AGE). In several simulated gastric fluid experiments, roasted peanut proteins manifested stronger resistance to digestion than both raw and boiled proteins [55]. Mass spectrometry showed that even when intestinal fluid was added to the simulated gastro-intestinal/duodenal fluid experiment, Ara h 1 persisted and appeared as a 65 kDa on the gel in both reducing and non-reducing conditions [57]. Ara h 2 obtained an anti-trypsin digestibility [58] and lasted a longer time on the SDS–PAGE than the raw peanuts when exposed to SGF [54] and even simulated gastric digestion, regardless of the electrophoretic conditions [57]. This idea was endorsed by other experiments in which some of the peanut allergens, and more prominently Ara h 2, resisted simulated oral–gastroduodenal digestion in raw [65] and roasted peanuts [66]. The ability of allergens’ penetration into the body and absorbance through the GI, specifically the intestines, was highly improved after roasting. Caco-2 cells, a line derived from colon carcinoma with typical abilities of absorbance of molecules as human enterocytes in the intestines, are used to assess the differences in the allergens’ capabilities of penetration after heat processing [67]. The conducted experiment proved that the uptake of Ara h 3 extensively increased after roasting upon viewing the inhibition of cell viability and proliferation. The aggregation of the allergen into a complex polymer with more recognizable epitopes qualified it to be more readily absorbed by cells, increasing its effect on the host. The same applies to the internalization of allergens into human monocyte-derived dendritic cells (MDDCs). As internalization of allergens is time- and dose-dependent, the experiment presented quantitative proof that, at every point measured, the penetration of roasted Ara h 2 was much higher than raw Ara h 2, reaching a maximum at 2 h of incubation [61]. Ara h 3 was internalized similarly to Ara h 2, primarily via the mannose receptors, but in greater quantities. The chemical modifications commonly produced during roasting, such as AGEs and advanced lipoxidation end-products (ALEs), could be a major factor that explains dominance in the internalization of the major allergens into the cells [68].

### 4.3. Microwaving

Microwaving is the processing of food materials by means of electromagnetic waves, creating multiple changes at the sample–wave interface. The process is characterized by an uneven heat distribution due to the positional effect of the sample in the microwave oven. It can be used for many purposes in food processing, such as baking, defrosting, pasteurization, and heating. Recent studies assess the role of this electromagnetic treatment in mitigating the allergenicity of several foods, including peanuts. Microwaved peanuts showed a 54% decrease in their total protein content [58]. The molecular analysis of the extracted proteins exhibited an insoluble aggregate of high-molecular-weight proteins, whose formation is proportional to the processing time. However, the IgE-binding capacity of proteins obtained from microwaved peanuts remained high, especially in the case of Ara h 2. The reactivity of the 19 kDa and 17 kDa isoforms of Ara h 2 was retained by 71% and 59%, respectively, taking raw peanuts as a reference [58]. This may be due to irregularities of the heating process or an insufficient duration of exposure to the treatment.

### 4.4. Ultrasonication

Ultrasonication is classified under the novel processing methods applied to food materials. It consists of irradiating food samples with high-energy ultrasonic waves, resulting in physical and chemical modifications. The interaction between the waves and the food samples results in the formation/collapse of bubbles within the medium. The sudden decompression of these bubbles creates pressure and temperature gradients and generates high shear energy waves in treated materials, leading to the alteration of their internal composition [69]. The impact of this constraint on food matrices is proportional to the treatment power and frequency. These events impose several structural changes on the resident macromolecules, including proteins. In fact, ultrasonication-induced matrix decompression is shown to affect hydrogen bond formation, increasing the susceptibility of proteins to unfolding and cleavage. It also impacts protein-to-protein interactions due to the alteration of protein conformation [19]. The treatment of roasted peanuts by ultrasound waves resulted in increased protein solubility and induced peptide bond cleavage. Moreover, ultrasound treatment resulted in a significant drop in Ara h 1 levels of treated samples with respect to those of untreated roasted peanuts, although the levels of Ara h 2 were not markedly lowered after the treatment [70].

### 4.5. Frying

It is essential to differentiate between shallow and deep frying. Shallow frying is the introduction of a food into a thin hot oil layer, whereas deep frying is when the food is entirely submerged in the hot oil. It has been noted that shallow frying slightly decreased the immunogenic potential of some major allergens by causing extensive modifications to their structure. A 21% and 2% decrease in α-helices and β-sheets content in Ara h 1, respectively, were detected in the secondary structure, taking raw peanuts as a reference [56]. In the tertiary and quaternary structures, a remarkable change was observed in the occurrence of irregular coils and in the leap of approximately 13-fold in hydrophobic index. The inner hydrophobic residues that surfaced indicated the release of polar amino acids and cross-linking of the aromatic amino acids. This implies the aggregation, or, in other words, the decrease in solubility of the allergens that collectively affect the function of those proteins. This thermal treatment still needs further investigation, since there is no definitive conclusion as to whether it slightly decreases or increases the immunogenic potential of the allergens. Although a decrease of approximately 9.4% in the IgE-binding capacity was noted, high aggregation and rearrangement of Ara h 1 might protect the epitopes from losing their abilities or even form neoepitopes that can interact with the immune system, increasing the elicitation.

Regarding the deep frying, smearing of low-molecular-mass (10 kDa) fragments revealed that they gradually became darker as frying time increased, with the 8-minute result (about 1.72 g/100 g peanut) being the highest (1, 3, 6, and 8 min) [71,72]. At the maximum interval, the peanuts’ color changed to brown, and they were at risk of burning. Similarly, their analysis referred to this intensity increase as a decrease in solubility of the allergens. With all these factors combined, a decrease in allergen solubility of 66.5% after deep frying was observed. Moreover, according to the BCA assay, the total extractable deep-fried proteins were 15%, meaning they decreased by approximately 4.8-fold compared to the crude sample [58,71]. SDS–PAGE analysis revealed an increase in the intensity of previously soluble high-molecular-mass protein on the gel, and Western blot test confirmed this result, albeit with greater intensity decreasing peanuts Ig-E binding capacity as well [58,63,71]. The antibody capacity of Ara h 1 showed a remarkable decrease in its intensity. Similarly, Ara h 2 IgE-binding properties for 19 kDa and 17 kDa decreased in strength by 70% and 38% in 6 min of deep frying, respectively [58,71]. In other experiments using ELISA tests, Ara h 2 decreased by 50%, while Ara h 8 did not change within up to 6 min of deep frying. However, upon exceeding this time (6 min), when Maillard reactions occurred, the IgE-binding capacity of Ara h 2 was higher than that in raw peanuts, and even gained an anti-trypsin digestibility property [58]. This puts on the table a conflict that needs further research, on whether integrity-preserving deep frying does not alter or decrease the allergenicity. One certainty is that frying decreased degranulation-β-hexosaminidase release by up to 61.52% after digestion as compared to raw peanuts [60].

### 4.6. High-Pressure Steaming/Autoclaving

The use of steam under high pressure is one of the most common processing methods applied on peanuts. High-pressure steaming and dry autoclaving are two sides of the same coin, used interchangeably in the literature. Nonetheless, wet autoclaving is steaming following hydration of peanuts by presoaking them in ultrapure water. In a study investigating the effect of various thermal processing methods, the highest aggregation state and fading speed were recorded for pre-soaked high-pressure-steamed peanuts [58]. Interpretation of this observation might be correlated to overnight soaking, which increased water activity and contributed to the higher structural change. Unlike most studies, only few were skeptical about this conclusion, claiming that it could not be valid because of the presence of two variables, high pressure and heat treatment, along with the hydration factor [71]. It causes extensive fragmentation of allergens, as LC–MS/MS analysis detected a vast increase in the number of peptides. Like other treatments, more efficient wet autoclaving alters secondary and tertiary structures. It also increases the percentage of extended-sheet structures along the moiety [58,73]. This promotes the aggregation of large protein complexes along with a series of unfolding, crosslinking, and chemical fluctuation (such as glycosylation and targeted oxidation) episodes. Wet autoclaving induces modifications in lysine, cysteine (sulfide bridges), and arginine residues, thus altering solubility.

The total extractable proteins decreased by 30–40% after wet autoclaving. A 91%, 61%, 55%, and 60% decrease in peanut content was measured for Ara h 1, Ara h 2, Ara h 3, and Ara h 6, respectively, with Western blot and SDS–PAGE. Subsequently, IgE-binding capacity was remarkably diminished [72,74,75,76]. Others, however, claim that the protein content is unchanged, or slightly altered [75]. All aforementioned experiments agree that the number of peptides associated with allergens increased with much lower molecular mass and concentration, specifically Ara h 2 and Ara h 7 on SDS–PAGE [72,76]. LC-high-resolution–MS/MS analysis also showed that the digestibility of such allergens increased upon treatment exposure [71,75]. Moreover, allergen recovery after disappearance from the SDS–PAGE was approximately null after wet autoclaving. This builds on the hypothesis that a synergistic effect of hydration, heat, and pressure diminish allergen function.

Wet autoclaving ceased the anti-proliferative effect of the allergens in Caco-2 cells. The results of cell viability showed improvement after treatment. Presumably, the release of small bioactive peptides that boost growth and reduce the penetration of allergens into the cells justifies increased cell viability. The decreased stimulation of the immune cells (T cell activation and inflammatory mediators), the feeble and massively reduced IgE capacity, and the managed chemokines, cytokines, and growth factors reflect the decrease in polarization of the immune cells resulting in a milder immune response [72,75].

Without presoaking, etiology in humans was detected through SPT for raw and autoclaved peanuts. The effectiveness of autoclaving was revealed by the noticeably reduced wheal diameter (SPT) in patients suffering from mild oral symptoms [63,71]. This was not, however, the case for patients who had previously suffered from anaphylaxis. The same applies to autoclaved roasted peanuts. Structure change in autoclaved and autoclaved roasted peanuts was consistent with this conclusion. It was a result of oligomerization, degradation due to free radicals attack on the side chains and peptide fragments, and even reassociation and aggregation due to cross-linking and hydrophobic interaction of peanut allergens [58,62,63,71,72].

Although complete degradation of Ara h 2, Ara h 7, and Ara h 8 and reduction in Ara h 1 were observed, the resistance of some fragments of Ara h 3 was noted after 20 min of autoclaving, suggesting that some peanut proteins might not be sufficiently susceptible to this treatment [58,63,72]. As for the protein content, SDS–PAGE and Western blot assessments showed that Ara h 1 monomer band faded proportionally to the processing time. The total extractable allergen content in raw peanuts decreased from 72% to 25% in steaming [58,71]. As we explained for previous treatments, these changes collectively cause a decrease in solubility as supported by the Bradford method measurements. Ara h 1 and Ara h 2 content were nearly null, less than 0.1 g and 0.2 g per 100 g, respectively [71]. On the other hand, at short cooking time (1 min), the three-dimensional structure of the Ara h 1 and Ara h 2 that is rich in disulfide bonds was slightly and insufficiently altered, and hence incapable of causing a change in resistance, solubility, or function [63].

Although normal steaming made changes in the characteristics of the allergens, its IgE antibody reactivity was still surprisingly strong. On the contrary, the case was different with mild steaming, where lower IgE binding capacity (75%) for all major allergens was recorded when compared with crude peanuts. In the case of harsher treatments, such as steaming for more than 20 min, the IgE capacity remarkably decreased, the whole peanut was rendered soft, and its structural integrity was deformed [58,71]. Lastly, study of human-like enterocytes (Caco-2 cells) manifested an inhibition in the allergen anti-proliferative effect upon autoclaving and improved their viability up to 49.3%. The notable decrease in IgE capacity is rooted to all the characteristics altered by autoclaving.

Finally, instant controlled drop (DIC) is a technique based on processing samples under high steam pressure. In this regard, it resembles autoclaving, except in the last step where the DIC is ended with an abrupt depressurization to the vacuum. This final drop to the vacuum induces partial water vaporization from the treated sample and its cooling, thus preventing further heat damage. DIC has been tested on peanuts, given the well-known effects of pressure-based treatments on peanut allergenicity. Some studies reported that DIC treatment is associated with an increase in the total crude protein content, assessed after defatting, but clarified that mild treatment conditions applied over whole raw peanuts did not significantly impact their protein profile epitopes and the corresponding immunoreactivity [77,78,79]. The effect of DIC on peanut crude protein content and immunoreactivity was directly proportional to the severity of treatment conditions in terms of pressure and time of treatment, unlike bioavailability, which declined with the intensification of the conditions. SDS–PAGE and IgE immunoblots of raw and roasted peanuts displayed similar band patterns before and after DIC [78]. However, previous studies reported that increasing the treatment conditions has led to lighter bands on SDS–PAGE and IgE immunoblots [77]. This claim was supported by stating that DIC treatment at the same pressure and time conditions generates a remarkable reduction in 65 kDa protein bands and eliminates the immunoreaction of bands less than 20 kDa [77]. Further investigations may be needed on the matter since other sources presented results displaying the same pattern of IgE-reactive bands for roasted peanuts, before and after DIC treatment under hard conditions [79].

### 4.7. Factors Affecting the Experimental Results of Treatments

Before delving into details about the differences existing between the processing methods applied on peanuts, it is worth mentioning that intertwining factors interfere with the experimental results by affecting the extractability of allergens and their bioavailability. Consequently, a mere quantitative comparison between the obtained allergens is not an option. Even if the quantities happened to change, it is not definite that their potential and function are altered. Additionally, extraction methods play a chief role in the assessment of the allergenicity of raw and processed peanuts. Studies have shown that protein extraction methods have different efficiencies in peanut allergen recovery. A comparative study between various extraction methods revealed diverse contents of extracted major allergens, which is considered a modulating factor for the basic assessment of the allergenicity [76]. Thermal treatments are known to induce the formation of aggregates, the physical and chemical properties of which differ from those of the original allergens. Indeed, allergen aggregates and neoallergens display altered solubility, structure, and digestibility. In turn, this will affect their extractability in different buffers, ultimately impacting the overall allergenicity assessment [19,76]. Other treatment methods, such as boiling, are known for their causing a reduction in peanut protein content. Boiling induces the externalization of peanut proteins onto water, thus lowering the bioavailability of these proteins and the allergenicity of the nuts as well [57]. In addition, defatting may alter protein extractability under different conditions and should be accounted for in analysis. It was shown that protein extracts obtained from non-defatted, thermally treated peanuts have low concentrations as compared to those of defatted peanuts [57,58]. The reason behind this difference is attributed to the lowered solubility of protein aggregates, formed by the Maillard reaction that takes place especially during thermal treatment. Finally, simulated digestion of the proteins obtained from different extracts varied, with the digestibility of raw peanut proteins being the highest among other extracts. Enzymes are functional over raw peanut proteins since they retain their original characteristics. Nonetheless, when it comes to the processed altered structure, a different collection of molecules surfacing is subjected to enzymes [57]. Accordingly, digestion efficiency is another factor that is thought to affect protein yields during extraction. In short, to attain adequate and significant results that are comparable between crude and processing or among different processing methods, a kinetic analysis should be conducted considering the interplay between the variables encountered.

### 4.8. Comparison between all Processing Methods

In general, thermomechanical treatments of peanuts are known as having a more or less important impact over protein content, structure, solubility, and immunogenicity of the allergens. This is reflected by a decrease in hydrophilic proteins due to their aggregation, accompanied by an increased intensity of hydrophobic protein. Several factors play a role in determining the severity of the thermal impact, including the operating conditions such as the time of treatment, the temperature, or the pressure applied.

Based on previous studies, wet processing proved its greater efficiency over dry processing. Using boiling and steaming, which are classified as wet methods, Ara h 1, Ara h 2, and Ara h 3 monomers showed a massive decrease in their intensity, steeper than what was observed with dry methods such as microwaving, roasting, and frying. Steaming had the highest record of the smeared bands, with low allergen solubility and IgE-binding capacity. This may be due to the thoroughness of heat penetration due to the high pressure [58,71]. Comparison of wet and dry autoclaving corroborated the claim of the higher efficiency of hydration over its absence; major and minor allergens were much lower. Fragmentation was extremely prominent in wet autoclaving as compared to dry autoclaving. A 55% elevation was observed in the number of peptides for major and minor allergens with wet autoclaving in comparison to raw peanuts, whereas a much lower fragmentation of 4–20% occurred when autoclaving was applied solely. Protein recovery was subtly recorded after dry autoclaving, but totally absent after wet autoclaving. Furthermore, an unprecedented, enhanced reduction in the protein content was observed when a hydration step was added to the protocol [72,75]. Last but not least, statistical Tukey–Kramer tests showed that a lower IgE-binding to the human plasma antibodies was recorded in dry autoclaving [72]. Structural alteration was more obvious in wet processing treatment, and the effect was even enhanced when pressure was added.

On the other hand, there was a differentiation between roasting and boiling on the SDS–PAGE where roasting mimicked the results of the raw peanuts, but boiled peanuts manifested weaker bands of some major allergens [54]. However, upon the addition of the digesting enzyme with amylase in simulated salivary fluid SSF, fewer peanut proteins were expressed in the gels of both treatments compared to the raw peanut chew sample [80]. One logical speculation could be that heat unstable proteins have been degraded, so fewer aggregations are released to soluble fractions. In all processed peanuts, fragments corresponding to Ara h 1 and Ara h 3, soluble fractions were susceptible to digestion, unlike those of Ara h 2 and Ara h 6 that were poorly digested after 120 min of pepsin exposure [60]. The intensity of boiled peanut proteins faded faster after digestion than that of roasted peanuts. Moreover, analysis of mass spectrometry of the insoluble fraction detected that there was much more aggregation left-over of Ara h 2 after the digestion time was completed in roasted peanuts when compared to the boiled peanuts. Unlike the soluble fraction, there was a faint band in the insoluble fraction after the duodenal digestion of roasted peanuts. Rabbit anti-allergen immunoblotting analysis was consistent with this conclusion [80]. Upon gastric digestion, plasma of soluble fractions indicated the presence of Ara h 2, Ara h 3, Ara h 6, and Ara h 7. The immunoreactivity of allergens’ proteolytic fragments was the lowest for boiled, followed by raw and roasted in ascending order. Unlike roasted peanuts, the allergens found in boiled peanuts’ immunoblot manifested very weak, nearly nonexistent IgE-binding capacity. However, that of roasted peanuts recorded a binding capacity even greater than that of raw peanuts [80]. The difference is significant and steeper between roasted and boiled after the intestinal digestion. According to densitometry [57], the digestion efficacy of roasted peanuts was lower than that of boiled peanuts during this phase. To further investigate the differences between roasting and boiling, an in vitro experiment was performed on the RBL-2H3 cell line, which resembles mast and basophilic cells in vivo [55,60]. This cell can bind to immunoglobulins and release inflammatory mediators such as histamine and β-HXA. Roasted peanut mice produced several times more β-HXA than raw or boiled peanuts [55]. This reinforces the postulation that roasting acts as a hyperallergic factor, whereas boiling could be a potentially hypoallergenic procedure.

## 5. Conclusions

During the last decade, peanut consumption has risen to unprecedented values per capita. Mitigation of peanut allergenicity has the potential to be a game changer for allergic individuals. Development of trivial and novel processing methods has become a necessity not only for scientists, but also for food industrialists, to impose structural and/or chemical modifications on allergens. Although none of the existing methods is qualified as fully effective in eliminating the allergenic potency of peanut proteins, the methods combined with a hydration step proved their higher efficacy. From this viewpoint, consuming wet autoclaved peanuts could be a promising alternative for susceptible individuals. Water penetration within the peanut matrix under pressure may result in a softer texture associated with a higher efficiency in allergenicity mitigation. Likewise, boiling showed a decreased level of peanut allergens. However, boiled peanuts retained some of their allergenic properties due to formation of neoallergens during the treatment. Lastly, deep frying of peanuts during 6 min resulted in reducing their allergenicity. The limitation of this process resides in the color and taste alterations following longer treatment times.

To conclude, it is fundamental to unveil the mechanistic details of primary peanut sensitization in order to fully understand peanut allergenicity [24]. Further studies should be directed towards the optimization of all of these techniques in order to ensure a sustainable level of hypoallergenicity in the treated peanuts, while preferably preserving their structural integrity and nutritional value. More importantly, the digestibility of modified allergens must be investigated to fully assess their ultimate allergic potential once consumed by peanut-allergic individuals.

## Figures and Tables

**Table 1:** Physicochemical properties of peanut allergens.

Allergen Superfamily		Allergen Name	Molecular Weight (in kDa)	Resistance to Heat and Digestion	Interaction with Water
Cupin (Vicilin-type, 7 S globulin)		Ara h 1	64	Resistant	Hydrophilic
Prolamin (2 S albumin–conglutin)		Ara h 2	17	Resistant	Hydrophilic
		Ara h 6	15	Resistant	
		Ara h 7	15	-	
Cupin (11 S globulin–glycinin)		Ara h 3.01	60	Resistant	Hydrophilic
		Ara h 3.02	37 (fragment)		
Profilin		Ara h 5	15	Minimal resistance	Amphipathic
Bet v 1 (PR-10 protein)		Ara h 8	17	Minimal resistance	Hydrophilic
Prolamin (Non-specific lipid-transfer protein nsLTP)	Type 1	Ara h 9	9.8	Resistant	Hydrophobic
	Type 2	Ara h 16	8.5		
	Type 1	Ara h 17	11		
Glycosyl transferase (oleosin)		Ara h 10	16	Minimal resistance	Hydrophobic
		Ara h 11	14		
		Ara h 14	17.5		
		Ara h 15	17		
Scorpion toxin-like knottin (defensin)		Ara h 12	8 (reducing), 12 (non-reducing)	-	Hydrophilic
		Ara h 13	8 (reducing), 11 (non-reducing)		
Cyclophilin		Ara h 18 (pan-allergens)	21	Minimal resistance	Hydrophilic

**Table 2:** Functions of peanut allergens, cross-reactivity, and clinical relevance.

Allergen Name	Function	Cross-Reactivity	IgE-Binding Potential	Clinical Relevance
Ara h 1	Seed storage protein	Brazil nut, cashew, hazelnut, peanut, walnut, soybean, lupin, peas, *c*hickpea, lentil	33 to 65%	Severe systemic reaction up to anaphylaxis
Ara h 2		Brazil nut, cashew, hazelnut, walnut, soybean, *c*hickpea	42 to 100%	
Ara h 3.01 Ara h 3.02		Brazil nut, cashew, hazelnut, walnut, soybean, lupin, pea, *c*hickpea, lentil	16 to 50%	
Ara h 5	Regulator of cellular processes Actin-binding protein Transport across membrane Cytoskeletal dynamics	Brazil nut, cashew, hazelnut, walnut, soybean, lupin, lentil	3% to 24% (in birch pollen-allergic people)	No or local clinical reaction Pollen food allergy syndrome
Ara h 6	Seed storage protein	Soybean, *c*hickpea	85% to 92%	Severe systemic reaction up to anaphylaxis
Ara h 7		-	43% to 80%	-
Ara h 8	Stress mechanism Plant defense	Other PR-10 allergens, soybean	2.4% to 49% (in birch pollen-allergic people)	Local clinical reaction Mild oropharyngeal reaction
Ara h 9	Lipid transfer across membrane Stress mechanism Plant defense	Chestnut, almond, peach, *Rosaceae* family, pear, plum, cherry, strawberry, lentil, sunflower, bean, pea	-	Systemic reaction
Ara h 10	Structural proteins: oil bodies	Other soy and buck wheat group	-	Local clinical reaction
Ara h 11		-	-	-
Ara h 12	Plant defense	-	-	-
Ara h 13		-	-	-
Ara h 14	Structural proteins: oil bodies	-	-	-
Ara h 15		-	-	-
Ara h 16	Lipid transfer across the membrane Stress mechanism Plant defense	Pollen, olive pollen, most respiratory allergens	-	-
Ara h 17				
Ara h 18	Peptidyl–prolyl cis–trans isomerase	Pollen, olive pollen, most respiratory allergens	87% POS IgE-binding for r Ara h 18	Local and temporary clinical reaction

## Data Availability

Data is contained within the article.
